# A search to the target tissue in which RA-specific inflammation starts: a detailed MRI study to improve identification of RA-specific features in the phase of clinically suspect arthralgia

**DOI:** 10.1186/s13075-019-2002-z

**Published:** 2019-11-27

**Authors:** Xanthe M. E. Matthijssen, Fenne Wouters, Debbie M. Boeters, Aleid C. Boer, Yousra J. Dakkak, Ellis Niemantsverdriet, Annette H. M. van der Helm-van Mil

**Affiliations:** 1 Department of Rheumatology, Leiden University Medical Center, P.O. Box 9600, 2300 RC Leiden, The Netherlands; 2 Department of Rheumatology, Erasmus Medical Center, Rotterdam, The Netherlands

**Keywords:** Rheumatoid arthritis, MRI, Biomarkers, Cohort study, Risk factors

## Abstract

**Objective:**

Based on a unique cohort of clinically suspect arthralgia (CSA) patients, we analysed which combinations of MRI features at onset were predictive for rheumatoid arthritis (RA) development. This was done to increase our comprehension of locations of RA onset and improve the predictive accuracy of MRI in CSA.

**Methods:**

In the discovery cohort, 225 CSA patients were followed on clinical arthritis development. Contrast-enhanced 1.5 T MRIs were made of unilateral metacarpophalangeal (MCP) (2–5), wrist, and metatarsophalangeal (1–5) joints at baseline and scored for synovitis, tenosynovitis, and bone marrow edema. Severity, number, and combinations of locations (joint/tendon/bone) with subclinical inflammation were determined, with symptom-free controls of similar age category as reference. Cox regression was used for predictor selection. Predictive values were determined at 1 year follow-up. Results were validated in 209 CSA patients.

**Results:**

In both cohorts, 15% developed arthritis < 1 year. The multivariable Cox model selected presence of MCP-extensor peritendinitis (HR 4.38 (2.07–9.25)) and the number of locations with subclinical inflammation (1–2 locations HR 2.54 (1.11–5.82); ≥ 3 locations HR 3.75 (1.49–9.48)) as predictors. Severity and combinations of inflammatory lesions were not selected. Based on these variables, five risk categories were defined: no subclinical inflammation, 1–2 locations, or ≥ 3 locations, with or without MCP-extensor peritendinitis. Positive predictive values (PPVs) ranged 5% (lowest category; NPV 95%) to 67% (highest category). Similar findings were obtained in the validation cohort; PPVs ranged 4% (lowest category; NPV 96%) to 63% (highest category).

**Conclusion:**

Tenosynovitis, particularly MCP-extensor peritendinitis, is among the first tissues affected by RA. Incorporating this feature and number of locations with subclinical inflammation improved prediction making with PPVs up to 63–67%.

## Background

Since a decade, increasing attention is being paid to identify patients in ‘pre-rheumatoid arthritis’ stages, among which the symptomatic stage preceding clinical arthritis. This is done with the assumption that earlier identification of patients with (imminent) rheumatoid arthritis (RA) allows earlier intervention and thereby may result in better disease outcomes. This hypothesis is being evaluated in several ongoing proof-of-concept trials [1–4]. Currently, accurate risk stratification is crucial to include patients at high risk to enhance the power of these trials [5]; in the future, it might be valuable to prevent overtreatment as much as possible.

Risk stratification is optimal if both positive and negative predictive values (PPV, NPV) are high. Importantly, both values strongly depend on prior risks. The prior risk of developing arthritis in at-risk populations, either asymptomatic, such as healthy relatives of patients with RA, or symptomatic, is relatively low [6, 7]. Consequently, any test that is applied in an at-risk population easily reaches a high NPV but PPVs generally remain low. Patients with clinically suspect arthralgia (CSA) are considered to be at risk for progression to RA based on the clinical presentation according to their rheumatologists. Only ~ 8% of patients presenting with arthralgia at rheumatologic outpatient clinics are identified as having CSA, and these patients have, compared to the other arthralgia patients, a 55 times increased odds to develop RA [7]. This shows the accuracy of clinical expertise as first discriminator. Nonetheless, without further risk stratification, the absolute risk on RA development in this population is still moderate (~ 20%) [8]. Hence, other biomarkers are needed in patients with CSA to achieve accurate prediction making and high PPVs in particular.

Different types of biomarkers have been studied, among which are autoantibodies, markers of systemic inflammation, and subclinical joint inflammation [9, 10]. The presence of imaging-detected subclinical inflammation in hand and foot joints has been shown predictive for progression to RA in several studies, both when using ultrasound (US) or magnetic resonance imaging (MRI) [6, 8, 11]. Although less accessible, MRI has the advantages that it can depict bone marrow edema (BME) and is more sensitive and reproducible than US [12]. Previous studies have revealed that some degree of MRI-detected inflammation is also present in symptom-free persons of the general population, especially at higher age [13, 14]. The nature of these features is not completely elucidated, and degeneration may explain part of these findings. However, for diagnostic and prognostic purposes, it has been evidently shown that using asymptomatic persons as reference when defining a positive MRI decreased the number of false-positive results and increased the specificity and predictive accuracy of MRI [15]. We previously observed that patients with CSA and a positive MRI, i.e. inflammation more than this reference, have a risk of 31% to progress to RA during the next year. The NPV of a negative MRI was high (94%) [8].

Thus far, the predictive accuracy of MRI-detected subclinical inflammation in CSA has not been validated. Moreover, we hypothesized that presence of certain inflammatory MRI features could be associated with a higher risk on RA development. We therefore aimed to determine if the PPV of MRI can be improved by not only evaluating the presence of subclinical inflammation but also incorporating information on the severity, the number, and combinations of affected locations. We also aimed to validate the predictive accuracy of MRI in a separate set of patients with CSA. Finally, detailed studies on MRI predictors might also increase our understanding of the joint tissues that are first affected during RA development.

## Methods

### Patients

All patients studied were included in the Leiden CSA cohort, which has been described elsewhere [16]. In short, CSA patients had recent-onset (< 1 year) arthralgia of hand or foot joints and were considered at risk for progression to RA based on the clinical expertise of the rheumatologist. Per definition, CSA was not present if patients presented with clinical arthritis or if another explanation for the symptoms (e.g. osteoarthritis, fibromyalgia) was more likely. Furthermore, autoantibodies were rarely determined in primary care, in line with Dutch GP guidelines [17]. Hence, inclusion was mainly based on the clinical expertise (including pattern recognition) of rheumatologists. We have previously shown that the expertise of the rheumatologist is valuable in differentiating arthralgia patients [7].

The Leiden rheumatology outpatient clinic has close contact with GPs and early referral clinics to allow access to secondary care without delay [18]. This provided a unique setting to identify patients with joint symptoms at risk for RA development before clinical arthritis has developed. From all patients newly presenting with arthralgia, only a small percentage is identified as having CSA by rheumatologists [7]. Notably, the cohort was founded before the development of the EULAR definition of arthralgia suspicious for progression to RA, and fulfilment of this definition was not mandatory. MRI was made at baseline. Patients were prospectively followed with scheduled visits at 4, 12, and 24 months; additional visits were scheduled in case of increasing symptoms [16].

The Leiden CSA cohort was split in two datasets. Between April 2012 and April 2015, 241 patients with CSA were consecutively included; of these, 225 had a baseline MRI and were studied as discovery cohort. CSA patients presenting between April 2015 and September 2017 were evaluated for validation (*n* = 298). Patients that participated in a randomized double-blind proof-of-concept trial (50% treated with methotrexate, 50% with placebo) (*n* = 73) and patients without a MRI (*n* = 16) were excluded from the validation dataset (see the flowchart in Additional file 1). Hence, 209 CSA patients were studied for validation; baseline characteristics (age, sex, symptom duration, number of painful joints, CRP, autoantibody status) did not differ between patients with and without MRI (Additional file 1). Participation in the trial required presence of MRI-detected subclinical inflammation. There were no differences in baseline characteristics between eligible patients with subclinical inflammation that were included in the validation cohort and were excluded because of trial participation (Additional file 1).

### MRI

MRI with a musculoskeletal (MSK)-extreme 1.5-T MRI scanner (GE, Wisconsin, USA) was performed at baseline of metacarpophalangeal (MCP (2–5)), wrist, and metatarsophalangeal (MTP (1–5)) joints on the most painful side (dominant side in case of symmetric symptoms) < 1 week after the first visit to the outpatient clinic. A detailed scan and scoring protocol is provided in Additional file 1. MRIs were scored in line with RAMRIS by two readers blinded to clinical data [19, 20]. The interreader and intrareader ICCs were all > 0.90 (Additional file 1).

As done previously, an MRI was considered ‘positive’ when subclinical inflammation was present, meaning both readers scored inflammation (synovitis, BME or tenosynovitis) in ≥ 1 location that was present in < 5% of the healthy persons in the same age category at the same location [13, 15, 21]. Thus, since inflammation is scored semi-quantitively, it must be 1 RAMRIS point above the 95th percentile of healthy individuals of the same age group. Reference values were obtained from previous research in which we scanned 193 healthy volunteers of 3 age categories [13].

Patients and rheumatologists were blinded to all MRI data in the discovery cohort. In the validation cohort, presence/absence of MRI positivity was disclosed (because it determined eligibility for a double-blind proof-of-concept trial) but patients and rheumatologists remained blinded for any further detailed MRI data (such as on specific MRI features or locations).

### Outcome

The main outcome was development of clinically apparent inflammatory arthritis, objectified at physical examination by rheumatologists. None of the patients used DMARDs (including glucocorticoids) before arthritis development. The secondary outcome was development of RA, defined as clinical diagnosis plus fulfilment of the 1987 or the 2010 criteria for RA (ACPA-negative patients with diagnosis of RA have difficulties fulfilling the criteria as ≥ 11 involved joints are required, whereas ACPA-positive patients can fulfil the criteria with only 1 swollen joint [22–25]; to prevent a possible bias for ACPA-negative patients, patients that fulfilled the 1987 criteria were also classified as RA).

### Statistical analyses

#### MRI features studied to identify predictors

We aimed to investigate the severity, the number, and combinations of locations with subclinical inflammation. These MRI features were defined/selected as follows:

*Severity—*Severe subclinical inflammation was defined as 2 RAMRIS points scored by both readers above the reference described above.

*Number of locations with subclinical inflammation—*The number of locations (joint/bone/tendon) was counted and categorized after visual inspection of the Kaplan-Meier curves.

*Combinations of types and locations—*Since incorporating all possible combinations of lesions in standard analysis would cause significant risk of overfitting, we implemented three methods to search for potentially predictive combinations. Firstly, all possible pairs of MRI features were plotted and coloured according to their prevalence in converters and non-converters (no clinical arthritis development < 1 year); combinations that were visually potentially predictive were selected. Because presentation of raw data presentation is insightful, but also has disadvantages, all possible pairs of inflammatory MRI lesions were also studied with least absolute shrinkage and selection operator (LASSO) regression (lambda minimizing the 10-fold cross-validation error) [26]. Finally, principal component analysis (PCA), incorporating all inflammatory MRI features, was performed to find potentially predictive combinations composed of multiple MRI features. The first two components were considered as potential predictors.

#### Model derivation

The Kaplan-Meier curves and univariable Cox regression were used to study the candidate MRI variables with time until arthritis development as outcome. Significant predictors (< 0.05) were checked for collinearity with Pearson’s correlations (< 0.7), before performing multivariable Cox analyses. All candidate predictors were entered in the model, and backward selection was performed (*p* < 0.10). To confirm the selection of predictors, we also added the predictors in a LASSO regression model and studied how often they remained in the model in 1000 bootstrap replications [26]. Risk groups were made based on the identified predictors, and the observed 1-year risk of developing inflammatory arthritis was calculated in each of the risk groups with logistic regression. In these analyses, 1 year follow-up data were used; thus, patients that developed clinical arthritis after year 1 were categorized as non-convertors. Five patients (2.2%) were lost to follow-up in year 1 and considered as non-convertors. PPVs, NPVs, and area under the curve (AUC) were determined. Calibration was assessed with the Hosmer-Lemeshow test and a calibration graph.

#### Validation

We used the model of the discovery cohort to predict the 1-year survival probabilities of the individuals in the validation cohort and validated the PPVs in the validation cohort. Calibration and predictive values were assessed similar to the discovery cohort. Eight patients (3.8%) were lost to follow-up in the first year and considered as non-convertors.

Patients in the validation cohort with a positive MRI who participated in a randomized double-blind trial were excluded. Exclusion of part of eligible patients with a positive MRI (which is associated with arthritis development) could affect the rate of arthritis development in the validation cohort. We therefore accounted for MRI positivity by including the number of locations (0 = negative MRI; 1–2 or ≥ 3 = positive MRI) in all multivariable models. Other characteristics of the patients with subclinical inflammation that were included and excluded from the validation cohort were similar (Additional file 1); therefore, adjustment for MRI positivity is sufficient to adjust for the lower number of patients with positive MRI in the validation set. This is extensively explained in Additional file 1.

#### Sensitivity analyses

Predictive values were verified with the outcome inflammatory arthritis after 2 years in patients that were included 2 years before data extraction.

Also, predictive values were assessed in the subgroup of CSA patients that also fulfilled the EULAR definition of arthralgia suspicious for progression to RA, as this is a more homogeneous subset of patients, with a slightly higher risk for RA [27, 28].

Predictive values were also assessed for the secondary outcome, development of RA.

Analyses were performed using SPSS 23 and R 3.5.0. *p* values < 0.05 were considered significant.

## Results

### Baseline characteristics

Baseline characteristics are shown in Table 1. Characteristics of both cohorts were similar, except for a lower frequency of MRI positivity in the validation cohort (51% versus 35%; *p* = 0.002).

**Table 1:** Baseline clinical and MRI characteristics of patients included in the discovery and validation cohorts

	Discovery cohort (*n* = 225)	Validation cohort (*n* = 209)	*p* value
Age in years, mean (SD)	44 (13)	43 (12)	0.26
Female, *n* (%)	174 (77)	165 (79)	0.77
Symptom duration in weeks, med (IQR)	17 (9–32)	20 (9–44)	0.28
Localisation of initial symptoms			0.39
Small joints, *n* (%)	189 (84)	165 (79)	
Small and large joints, *n* (%)	22 (10)	26 (13)	
Large joints, *n* (%)	13 (6)	17 (8)	
Localisation of initial symptoms			0.76
Upper extremities, *n* (%)	162 (72)	134 (70)	
Upper and lower extremities, *n* (%)	39 (17)	34 (18)	
Lower extremities, *n* (%)	23 (10)	24 (13)	
Symmetrical localisation of initial symptoms, *n* (%)	166 (74)	127 (70)	0.35
Morning stiffness ≥ 60 min, *n* (%)	72 (36)	62 (34)	0.83
68-TJC, med (IQR)	6 (3–10)	5 (2–10)	0.23
Fulfilling the EULAR definition of CSA, *n* (%)	153 (68)	131 (63)	0.29
CRP level in mg/L, med (IQR)	3 (3–5)	3 (3–4)	0.59
ESR level in mg/L, med (IQR)	6 (2–13)	6 (2–14)	0.12
RF, *n* (%)	46 (20)	41 (20)	0.92
ACPA, *n* (%)	28 (12)	30 (14)	0.66
MRI-detected presence of subclinical inflammation (MRI positivity), *n* (%)	114 (51)	74 (35)	0.002

*p* value: chi-square tests, Fishers’s exact tests, Student’s *t* tests, and Wilcoxon’s rank sum tests were applied as appropriately. *SD* standard deviation, *n* number of patients, *RA* rheumatoid arthritis, *med* median, *IQR* interquartile range, *EULAR* European League Against Rheumatism, *CSA* clinically suspect arthralgia, *BME* bone marrow edema, *min* minutes, *TJC* tender joint count, *CRP* C-reactive protein, *ESR* erythrocyte sedimentation rate, *RF* rheumatoid factor, *ACPA* anti-citrullinated protein antibody, *MRI* magnetic resonance imaging

### Discovery cohort

Within a median follow-up of 108 weeks (IQR 54–114), 42 patients progressed to clinical arthritis, and 34 (15%) did so within the first year.

### Identification of predictors

In univariable analysis, severe subclinical inflammation was predictive for inflammatory arthritis development (Table 2).

**Table 2:** Results of univariable and multivariable Cox regression in discovery cohort with clinically apparent inflammatory arthritis as outcome

	Univariable	Final model after backward selection
Number of locations with subclinical inflammation		
0 locations (negative MRI)	Ref	Ref
1 or 2 locations	3.14 (1.40–7.04)	2.54 (1.11–5.82)
3 or more locations	6.28 (2.77–14.2)	3.75 (1.49–9.48)
Severe subclinical inflammation*	3.34 (1.48–7.54)	–
MCP-extensor peritendinitis	7.85 (3.91–15.8)	4.38 (2.07–9.25)
Combination of inflammatory lesion in wrist and MTPs	2.19 (1.15–4.16)	–
PCA component 1	0.92 (0.88–0.96)	–
PCA component 2	0.93 (0.83–1.04)	–

*MCP* metacarpophalangeal, *MTP* metatarsophalangeal, *n* number of patients

*Severe subclinical inflammation: inflammation that is 2 RAMRIS points above the 95th percentile of inflammation observed in healthy volunteers in the same age category as published previously [13]. Further explanation in Additional file 1

With respect to the number of locations with subclinical inflammation, visual examination of the Kaplan-Meier analysis resulted in three subcategories: 0 locations with subclinical inflammation, 1–2 locations, and ≥ 3 locations (Additional file 1). As shown in Table 2, the number of locations was predictive for arthritis development.

**Fig. 1:**
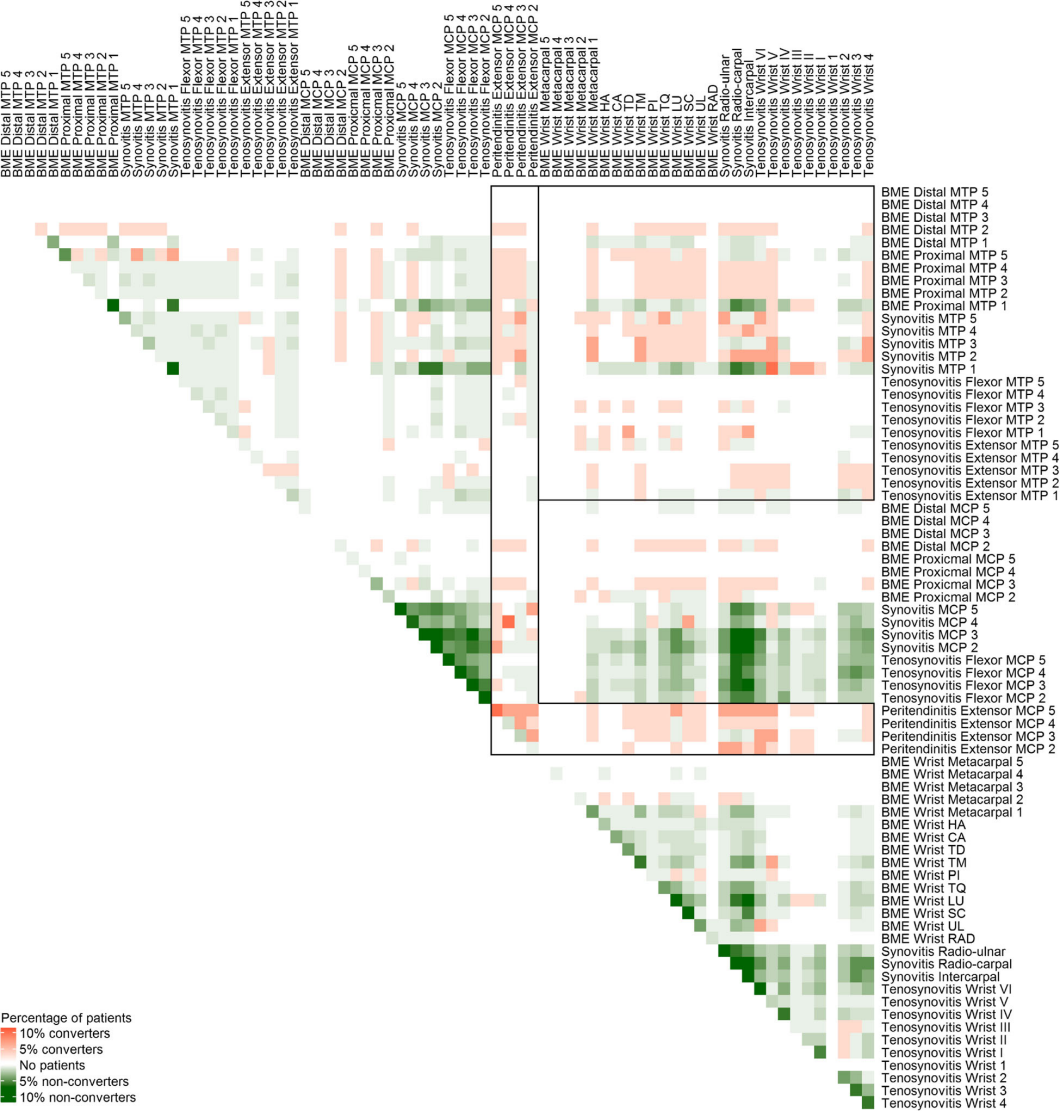
Plot of prevalence of all possible pairs of MRI inflammatory features in both converters and non-converters in the discovery cohort. Pairs of features that were only present in patients that progressed to arthritis < 1 year (converters; *n* = 34) and not in non-convertors (*n* = 191) are indicated in red. Pairs of features only present in non-convertors are indicated in green. The L-shaped box depicts extensor peritendinitis of the MCP (2–5) joints, and the rectangle depicts a combination of inflammation (synovitis, tenosynovitis or BME) in the wrist and in MTP (1–5). MRI, magnetic resonance imaging; CSA, clinically suspect artralgia; BME, bone marrow edema; MTP, metatarsophalangeal; MCP, metacarpophalangeal; HA, hamate; CA, capitate; TD, trapezoid; TM, trapezium; PI, pisiform; TQ, triquetrum; LU, lunate; SC, scaphoïd; UL, distal ulna; RAD, distal radius. Tenosynovitis wrist: (I) extensor pollicis brevis, abductor pollicis longus; (II) extensor carpi radialis brevis, extensor carpi radialis longus; (III) extensor pollicis longus; (IV) extensor digitorum communis, extensor indicus proprius; (V) extensor digiti quinti proprius; (VI) extensor carpi ulnaris; (1) flexor carpi ulnaris; (2) ulnar bursa, including flexor digitorum profundus and superficialis tendon quartets; (3) flexor pollicis longus in radial bursa; (4) flexor carpi radialis

Prevalence of all pairs of MRI features was plotted for patients with and without arthritis development ≤ 1 year (Fig. 1). Visual inspection suggested that a combination of inflammation in the wrist and in MTP joints was predictive for arthritis development. Additionally, all combinations with MCP-extensor peritendinitis, basically the presence of MCP-extensor peritendinitis, were potentially predictive. Therefore, the combination of inflammation in the wrist and in MTP joints and the presence of MCP-extensor peritendinitis were studied further. Both variables were indeed significant in univariable Cox regression (Table 2; Additional file 1).

LASSO regression using all possible pairs of inflammatory MRI lesions identified pairs that were very specific but present in few patients. Because most of these pairs were incorporated in the combination of wrist and MTP inflammation and MCP-extensor peritendinitis (Additional file 1), these latter were used in further analyses.

PCA was performed to search for patterns composed of multiple MRI lesions; this revealed no evident discrimination of patients with and without arthritis development. PCA component 1 was predictive for arthritis development, and PCA component 2 was not (Table 2; Additional file 1).

### Model derivation

**Fig. 2:**
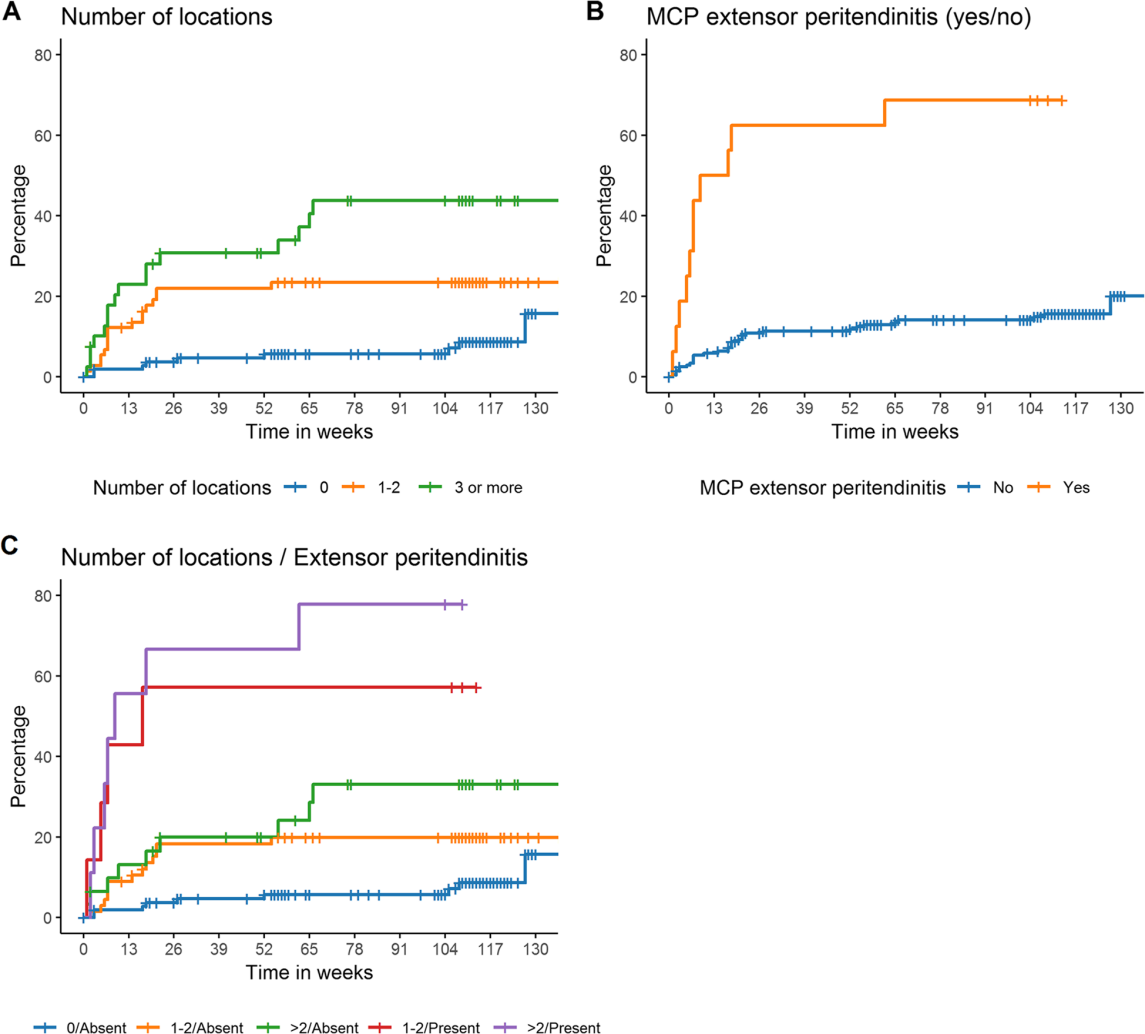
Kaplan-Meier curves showing the associations with inflammatory arthritis development for the number of locations with subclinical inflammation (**a**), presence of MCP-extensor peritendinitis (**b**), and both variables combined (**c**). 0/Absent: 0 locations with subclinical inflammation; no MCP-extensor peritendinitis. 1–2/Absent: 1–2 locations with subclinical inflammation; no MCP-extensor peritendinitis. > 2/Absent: 3 or more locations with subclinical inflammation; no MCP-extensor peritendinitis. 1–2/Present: 1–2 locations with subclinical inflammation; MCP-extensor peritendinitis. > 2/ Present: 3 or more locations with subclinical inflammation; MCP-extensor peritendinitis

Multivariable Cox regression of the five predictors revealed that number of locations and MCP-extensor peritendinitis were independently predictive, in contrast to severe subclinical inflammation, combination of an inflammatory lesion in wrist and MTPs, and PCA component 1 (Fig. 2; Table 2). LASSO regression in 1000 bootstrapped datasets confirmed that the number of locations (1–2 locations, 47%; ≥ 3, 61%) and MCP-extensor peritendinitis (91%) were selected more often than severe subclinical inflammation (45%), the combination of an inflammatory lesion in wrist and MTP joints (43%), and PCA component 1 (53%).

**Fig. 3:**
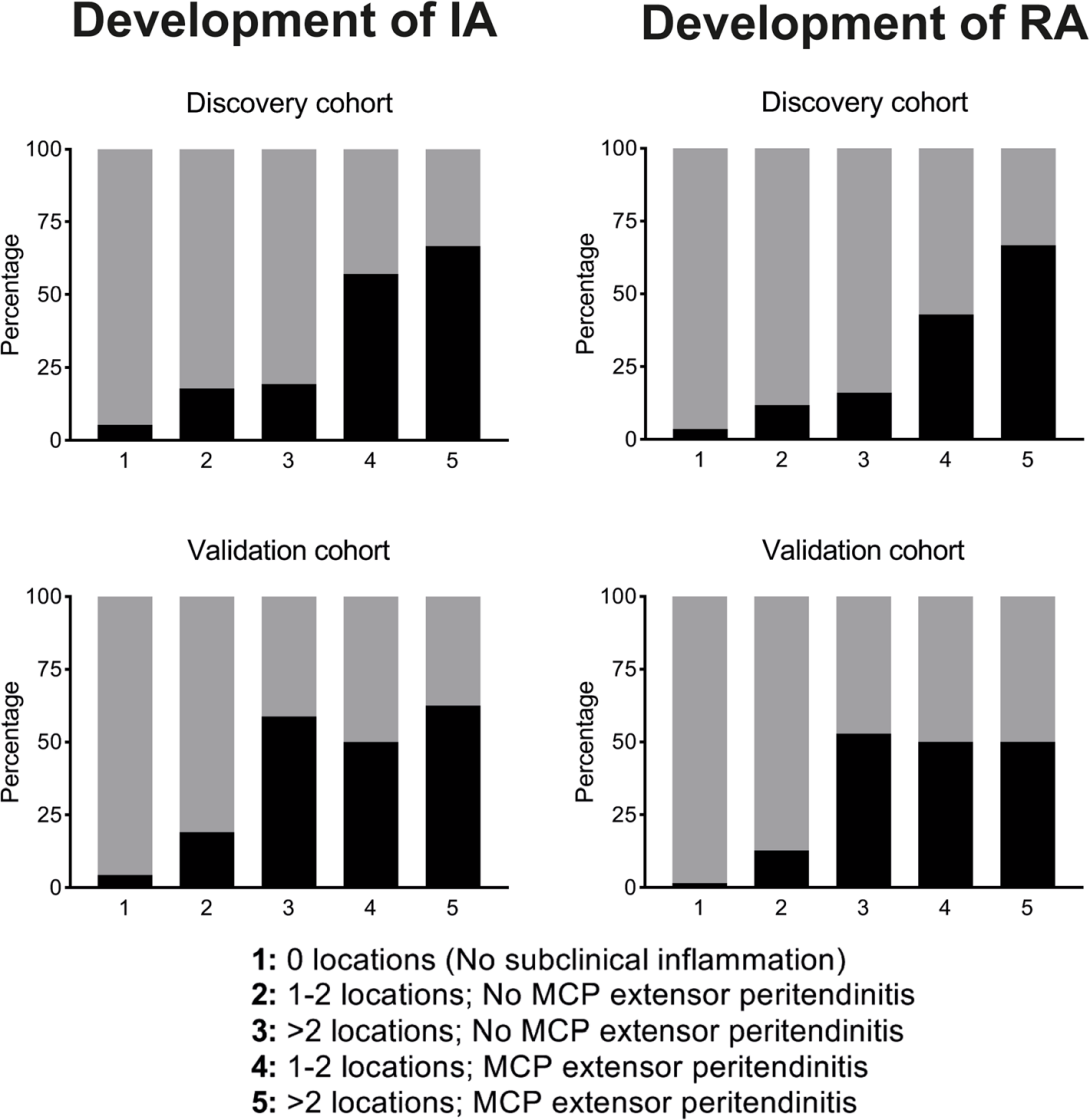
Observed proportion of patients that developed clinical apparent inflammatory arthritis and rheumatoid arthritis in the first year (PPVs in black) per risk category in the discovery and validation cohorts. IA, clinically apparent Inflammatory Arthritis; RA, rheumatoid arthritis; locations, number of locations with subclinical inflammation. Upper left graph: positive predictive values on IA in the discovery cohort; no subclinical inflammation (5% (95% confidence interval 3–11%, *n* = 111), 1–2 locations (18% (11–29%), *n* = 67), or ≥ 3 locations (19% (9–36%), *n* = 31) with subclinical inflammation but without MCP-extensor peritendinitis; and 1–2 locations (57% (25–84%), *n* = 7) or ≥ 3 locations (67% (35%–88%), *n* = 9) with MCP-extensor peritendinitis. Upper right graph: positive predictive values on RA in the discovery cohort; no subclinical inflammation (4% (95% CI 1–9%, *n* = 111), 1–2 locations (12% (6–22%), *n* = 67), or ≥ 3 locations (16% (7–33%), *n* = 31) with subclinical inflammation but without MCP-extensor peritendinitis; and 1–2 locations (43% (16–75%), *n* = 7) or ≥ 3 locations (67% (35–88%), *n* = 9) with MCP-extensor peritendinitis. Lower left graph: positive predictive values on IA in the validation cohort; no subclinical inflammation (4% (95% confidence interval 2–9%, *n* = 135), 1–2 locations (19% (10–33%), *n* = 47), or ≥ 3 locations (59% (35–78%), *n* = 17) with subclinical inflammation but without MCP-extensor peritendinitis; and 1–2 locations (50% (3–97%), *n* = 2) or ≥ 3 locations (63% (31–86%), *n* = 8) with MCP-extensor peritendinitis. Lower right graph: positive predictive values on RA in the validation cohort; no subclinical inflammation (1% (95% CI 0–5%, *n* = 135), 1–2 locations (13% (6–25%), *n* = 47), or ≥ 3 locations (53% (31–74%), *n* = 17) with subclinical inflammation but without MCP-extensor peritendinitis; and 1–2 locations (50% (3–97%), *n* = 2) or ≥ 3 locations (50% (22–78%), *n* = 8) with MCP-extensor peritendinitis

Based on the identified variables, patients were divided into five risk groups: no subclinical inflammation (‘negative MRI’), 1–2 and ≥ 3 locations of subclinical inflammation without MCP-extensor peritendinitis, and 1–2 and ≥ 3 locations with MCP-extensor peritendinitis. A form to calculate this risk score is presented in Additional file 1 and online [29]. Logistic regression predicted PPVs of arthritis development in the five risk categories of 5%, 18%, 20%, 60%, and 64%, respectively. The observed PPVs were as follows: 5%, 18%, 19%, 57%, and 67%, respectively. The NPV of no subclinical inflammation was 95% (Fig. 3). Predicted and observed conversion rates were plotted in a calibration graph (Additional file 1). The Hosmer-Lemeshow test showed good calibration (*p* = 0.92). The AUC was 0.74 (95% confidence interval 0.65–0.84). For comparison, a model that only considered MRI positivity/MRI negativity had an AUC of 0.69 (0.60–0.78) (Additional file 1).

### Validation

At 1 year, 15% (31/209) had developed arthritis. We validated the PPVs; the observed PPVs for arthritis development ≤ 1 year of the five risk categories were 4% (lowest risk category), 19%, 59%, 50%, and 63% (highest risk category), respectively (Fig. 3). The NPV of no subclinical inflammation was 96%. The AUC in the validation cohort was 0.81 (0.72–0.90) (Additional file 1).

The calibration plot (Additional file 1) shows good calibration, except in the group with ≥ 3 locations without MCP-extensor peritendinitis (predicted, 20%; observed, 59%; *n* = 17), yielding a significant Hosmer-Lemeshow test (*p* = 0.01).

### Sensitivity analyses

Predictive values were verified with the outcome inflammatory arthritis after 2 years follow-up. Slightly higher positive predictive values were obtained (Additional file 1).

Similar predictive values were obtained in the subgroup of CSA patients that also fulfilled the EULAR definition (discovery, *n* = 153; validation, *n* = 131; Additional file 1). Also, similar findings were obtained for RA development as outcome.

## Discussion

We aimed to increase the understanding of the tissues that are already subclinically inflamed preceding the development of clinical arthritis and observed that MCP-extensor peritendinitis is an early feature of RA. Moreover, we aimed to optimize the predictive value of information provided by MRI for clinical arthritis and RA development in patients presenting in secondary care with CSA. MCP-extensor peritendinitis and the number of locations with subclinical inflammation were independently predictive. Risk prediction of patients with a positive MRI was differentiated using these variables. Whereas patients with a positive MRI had, at group level, a PPV of 31% to develop RA during the next year [8], now a subgroup was found with a slightly lower risk (18–19%), but also subgroups with higher PPVs (up to 67%). The high NPV that was also observed previously was validated [8]. Importantly, this is the first study on the predictive accuracy of MRI in arthralgia that also demonstrated replication.

**Fig. 4:**
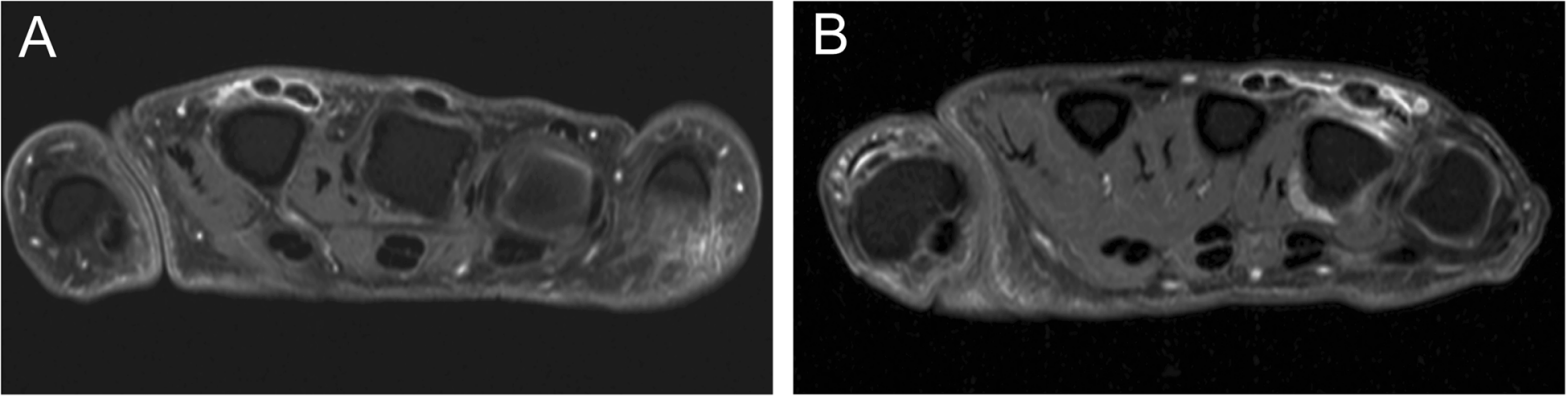
MRI examples of MCP-extensor peritendinitis. MCP-extensor peritendinitis in two CSA patients, depicted in T1-weighted FSE sequences with frequency selective fat saturation in the axial plane of the MCP joints after injection of gadolinium contrast. Patient A had extensor peritendinitis at the level of MCP 2. Patient B had extensor peritendinitis at MCP 4; this patient also had peritendinitis at the level of MCP 3 and synovitis at MCP 4 that was better visualized at adjacent slices

We observed that MCP-extensor peritendinitis (see Fig. 4 for an example) characteristically occurs before the development of clinical arthritis, in part of the RA patients. MCP-extensor peritendinitis is a relatively novel imaging finding, although several previous studies within classified RA showed that peritenditis of the MCP extensors (visualized by MRI or US) has a high specificity for RA [28, 29]. Whether involvement of this tendon occurs before or after other signs of inflammation (synovitis, osteitis) is unsolved, as longitudinal imaging data in the pre-arthritis phase of RA is scarce. Results of a recent study suggested that tenosynovitis of small joints in general was already increased at presentation with CSA, and preceded the development of osteitis and clinical arthritis, but further serial MRI studies are needed [30]. Whether micro-channels in the bare area of the joint are important in the spreading of inflammation is also a subject for further investigations.

The plantar side of the hand has been studied anatomically, and a tendon sheath at the level of MCP joints was found. The extensor side, however, is less extensively studied, but a tendon sheath here has not been documented evidently [31]. Therefore, the nature of the signal around the extensor tendons at the MCPs is as of yet unclear and is an interesting subject for further studies.

No validated scoring methods for MCP-extensor peritenditis exist; therefore, we adopted the method as proposed by Haavardsholm et al. [19]. Now that the relevance of this MRI finding has been shown, further development and validation of scoring methods are warranted.

This study made more efficient use of the information obtained by MRI. Nonetheless and not unexpectedly, the accuracy of MRI alone was moderate and can presumably be improved by adding other biomarkers (e.g. autoantibodies, markers of systemic inflammation). Ideally, AUCs and PPVs are obtained that are even higher than those observed here. Further research is needed to identify the best combination of biomarkers and validate this in independent datasets. Preferably, this will be performed in cohorts that are even larger in size than those studied here, so that sufficient predictors can be included in the model without overfitting the data.

A strength of this study is that results were validated in an independent dataset. Since we used a data-driven approach to find predictors, validation was essential for confirmation of findings. PPVs of the third risk category (≥ 3 locations, no MCP-extensor peritendinitis) differed in the two cohorts, possibly due to small sample sizes in this subgroup. Reassuringly, the PPV was higher in the validation cohort. Further validation is needed to more reliably determine the PPV of this subgroup.

Part of the patients eligible for the validation cohort had subclinical inflammation and participated in a RCT and were therefore excluded. Although this exclusion of patients with a higher risk of arthritis development will decrease the overall probability of arthritis development, correcting for MRI positivity ensures that within MRI categories, the predicted probabilities are still adequate (see Additional file 1).

Of note, 150 of the 225 patients in the discovery cohort were also included in a previously published analysis, which evaluated the association of a positive MRI with arthritis development [8]. The dataset at that time was insufficient to further evaluate separate inflammatory characteristics and to validate results.

A limitation is that in the first 77 of the 225 patients in the discovery cohort, contrast-enhanced and axial plane sequences were not performed in MTP joints (Additional file 1). Synovitis scoring without contrast is less specific [30]. Consequently, the number of locations with subclinical inflammation could be slightly overestimated in part of the discovery cohort. However, the PPVs of the number of locations were similar in the validation cohort, indicating that this effect seems limited.

Difference in follow-up duration between both cohorts could cause differences in effect sizes. Therefore, as all patients in both cohorts had ≥ 1 year follow-up, predictive values were determined at 1 year follow-up. This could have caused an underestimation of the conversion rates. More than 75% of patients in the discovery cohort converted to inflammatory arthritis < 1 year, as can also be seen in Fig. 2, indicating that somewhat higher PPVs can be expected when values would be determined after additional years of follow-up. This was indeed observed in the sensitivity analyses using 2 years of follow-up.

We used MRI to image subclinical joint inflammation. Although MRI is more sensitive than US, especially in the pre-arthritis phase [31], it is less feasible and more costly. This might currently hamper implementation of MRI in clinical practice in some centres or countries. Alternatively, in other centres or regions, MRIs are already made to search for subclinical joint inflammation and the data presented here allow evidence-based use of the data provided by MRI.

In conclusion, tenosynovitis, particularly MCP-extensor peritendinitis, is among the first tissues affected by RA. Incorporation of this feature and number of locations with subclinical inflammation improved prediction making for subgroups of patients, compared to MRI positivity/MRI negativity. These data allow evidence-based use of MRI in patients presenting with CSA to predict RA development. Further research is now needed to combine the present MRI data with other biomarkers to further improve risk stratification. Ultimately, this may reduce the possible risk of overtreatment of patients at risk for RA.

## Supplementary information

**Additional file 1.** Supplementary files.

## Data Availability

The datasets used and/or analysed during the current study are available from the corresponding author on reasonable request.

## Abbreviations

AUC: Area under the curve
BME: Bone marrow edema
CSA: Clinically suspect arthralgia
LASSO: Least absolute shrinkage and selection operator
MCP: Metacarpophalangeal
MRI: Magnetic resonance imaging
MSK: Musculoskeletal
MTP: Metatarsophalangeal
NPV: Negative predictive value
PCA: Principal component analysis
PPV: Positive predictive value
RA: Rheumatoid arthritis
US: Ultrasound
