# Supplementary material for: A search to the target tissue in which RA-specific inflammation starts: a detailed MRI study to improve identification of RA-specific features in the phase of clinically suspect arthralgia
Source: Arthritis Res Ther. 2019 Nov 27;21:249. doi: 10.1186/s13075-019-2002-z (PMC6880566; doi:10.1186/s13075-019-2002-z)
Supplement: Supplementary file 1 — Additional file 1. Supplementary files. [file 13075_2019_2002_MOESM1_ESM.docx]

# SUPPLEMENTARY FILE 1

# Patient flowcharts

# Patient selection flowchart - Discovery cohort

Patients included in Leiden CSA cohort between April 2012 and April 2015, (n=241)

No MRI data available:
- MRI contra-indication (n=4)
- Arthritis before MRI (n=1)
- Refused MRI (n=11)

Patients studied in discovery cohort with complete MRI data (n=225)

# Patient selection flowchart - Validation cohort

Patients included in Leiden CSA cohort between April 2015 and September 2017, (n=298)

No MRI data available:
- MRI contra-indication (n=6)
- Arthritis before MRI (n=1)
- Refused MRI (n=9)

Inclusion in double-blind placebo controlled randomized trial, no follow-up in CSA (n=73)

Patients studied in validation cohort with complete MRI data (n=209)

# SUPPLEMENTARY FILE 2

# Baseline characteristics of patients with or without a MRI

|  | MRI present in discovery cohort  (n=225) | No MRI in discovery cohort  (n=16) | p-value |
| --- | --- | --- | --- |
| Age in years, mean (SD) | 44 (13) | 46 (11) | 0.35 |
| Female, n (%) | 174 (77) | 13 (81) | 1.00 |
| Symptom duration in weeks, med (IQR) | 17 (9-32) | 17 (11-25) | 0.78 |
| 68-TJC, med (IQR) | 6 (3-10) | 6 (3-11) | 0.48 |
| CRP-level in mg/L, med (IQR) | 3 (3-5) | 3 (3-5) | 0.41 |
| RF, n (%) | 46 (20) | 5 (31) | 0.34 |
| ACPA, n (%) | 28 (12) | 4 (25) | 0.24 |

**Baseline characteristics of patients with or without a MRI in discovery cohort**

**Legend:** p-value: Chi-square tests, Fishers’s exact tests, Student’s t-tests and Wilcoxon’s rank sum tests were applied as appropriately. SD: Standard deviation; n:number of patients; med: median;

IQR: interquartile range; TJC: Tender joint count; CRP: C-reactive protein; RF: Rheumatoid factor; ACPA: Anti-citrullinated protein antibody

|  | MRI present in validation cohort  (n=209) | No MRI in validation cohort  (n=16) | p-value |
| --- | --- | --- | --- |
| Age in years, mean (SD) | 43 (12) | 40 (12) | 0.37 |
| Female, n (%) | 165 (79) | 13 (81) | 1.00 |
| Symptom duration in weeks, med (IQR) | 20 (9-44) | 14 (9-53) | 0.73 |
| 68-TJC, med (IQR) | 5 (2-10) | 5 (2-6) | 0.45 |
| CRP-level in mg/L, med (IQR) | 3 (3-4) | 3 (3-6) | 0.93 |
| RF, n (%) | 41 (20) | 1 (6) | 1.00 |
| ACPA, n (%) | 30 (14) | 3 (19) | 0.70 |

**Baseline characteristics of patients with or without a MRI in validation cohort**

**Legend:** p-value: Chi-square tests, Fishers’s exact tests, Student’s t-tests and Wilcoxon’s rank sum tests were applied as appropriately. SD: Standard deviation; n:number of patients; med: median;

IQR: interquartile range; TJC: Tender joint count; CRP: C-reactive protein; RF: Rheumatoid factor; ACPA: Anti-citrullinated protein antibody

# SUPPLEMENTARY FILE 3

# Patients excluded from validation cohort

|  | Patients with subclinical inflammation excluded from the validation cohort  (n=73) | Patients with subclinical inflammation included in the validation cohort  (n=74) | p-value |
| --- | --- | --- | --- |
| Age in years, mean (SD) | 47 (13) | 44 (13) | 0.19 |
| Female, n (%) | 43 (59) | 55 (74) | 0.07 |
| Symptom duration in weeks, med (IQR) | 17 (6-34) | 17 (7-35) | 0.66 |
| 68-TJC, med (IQR) | 4 (2-6) | 4 (2-10) | 0.46 |
| CRP-level in mg/L, med (IQR) | 3 (3-6) | 3 (3-6) | 0.92 |
| RF, n (%) | 20 (27) | 21 (28) | 1.00 |
| ACPA, n (%) | 14 (19) | 22 (30) | 0.20 |
| Studied predictors |  |  |  |
| Extensor peritendinitis in the MCP joints, n (%) | 8 (11) | 10 (14) | 0.83 |
| Presence of severe subclinical inflammation, n (%) | 6 (8) | 12 (16) | 0.22 |
| Number of locations with subclinical inflammation |  |  | 0.95 |
| 0 locations (negative MRI), n (%) | 0 (0) | 0 (0) |  |
| 1 or 2 locations, n (%) | 47 (64) | 49 (66) |  |
| 3 or more locations, n (%) | 26 (36) | 25 (34) |  |

**Baseline characteristics of patients eligible for the validation cohort but were excluded because of participation in a trial, compared to patients included in the validation cohort with subclinical inflammation**

**Legend:** p-value: Chi-square tests, Fishers’s exact tests, Student’s t-tests and Wilcoxon’s rank sum tests were applied as appropriately. SD: Standard deviation; n:number of patients; med: median;

IQR: interquartile range; TJC: Tender joint count; CRP: C-reactive protein; RF: Rheumatoid factor; ACPA: Anti-citrullinated protein antibody

# SUPPLEMENTARY FILE 4

# MRI scanning and scoring

# MRI scanning

MRI was performed on a MSK-extreme 1.5T extremity MRI system (GE, Wisconsin, USA) using a 145mm coil for the foot and a 100mm coil for the hand. The patient was positioned in a chair beside the scanner, with the hand or foot fixed in the coil with cushions.

In the hand (MCP2-5 and wrist) the following sequence was acquired before contrast administration: T1-weighted fast spin-echo (FSE) sequence in the coronal plane (repetition time (TR) 575 ms, echo time (TE) 11.2 ms, acquisition matrix 388×288, echo train length (ETL) 2). After intravenous injection of gadolinium contrast (gadoteric acid, Guerbet, Paris, France, standard dose of 0.1 mmol/kg) the following sequences were obtained: T1-weighted FSE sequence with frequency selective fat saturation (fatsat) in the coronal plane (TR/TE 700/9.7ms, acquisition matrix 364×224, ETL 2), T1-weighted FSE fatsat sequence in the axial plane (wrist: TR/TE 540/7.7 ms; acquisition matrix 320x192; ETL 2 and MCP-joints: TR/TE 570/7.7 ms; acquisition matrix 320x192; ETL 2).

The obtained sequences of the forefoot (MTP1-5 joints) were for the first 77 patients before contrast administration: T1-weighted FSE sequence in the axial plane (TR/TE 650/17ms; acquisition matrix 388x288, ETL 2); and T2-weighted FSE fatsat sequence in the axial plane (TR/TE 3000/61.8; acquisition matrix 300x224, ETL 7). Imaging of the foot was initially limited to pre-contrast axial sequences. For the latter 357 patients post-contrast sequences were included: T1-weighted FSE fatsat sequence in the axial plane (TR/TE 700/9.5ms; acquisition matrix 364x224, ETL 2) and: T1-weighted FSE fatsat sequence in the coronal plane (perpendicular to the axis of the metatarsals) (TR/TE 540/7.5ms; acquisition matrix 320x192, ETL 2). Field-of-view was 100mm for the hand and 140mm for the foot. Coronal sequences of the hand had 18 slices with a slice thickness of 2mm and a slice gap of 0.2mm. Coronal sequences of the foot had 20 slices with a slice thickness of 3mm and a slice gap of 0.3mm. All axial sequences had a slice thickness of 3mm and a slice gap of 0.3mm with 20 slices for the wrist, 16 for the metacarpophalangeal-joints and 14 for the foot.

We used the contrast enhanced T1-weighted fat suppressed sequence to assess BME in the MCP-joints of all patients. In the MTP-joints BME was assessed on T2-weighted fatsat sequences in the first 77 patients in the discovery cohort and on the contrast enhanced T1-weighted fat suppressed sequence in the latter 148 patients and in the validation cohort. According to the RAMRIS-method, T2-weighted fat suppressed sequences, or when this sequence is not available a short tau inversion recovery (STIR) sequence, should be used to assess BME. However, three previous studies have demonstrated that a contrast enhanced T1-weigthed fat suppressed sequence has a strong correlation with T2-weighted fat suppressed sequences (1-4). Furthermore, the arthritis subcommittee of the European Society of Musculoskeletal Radiology (ESSR) also recommends the use of contrast enhanced T1-weighted fat suppressed sequences for depicting BME (5). The T2-weighted image shows increased water signal and a contrast-enhanced T1-weighted sequence shows increased water content and the increased perfusion and interstitial leakage. A strong correlation has been shown in arthritis patients and in patients without inflammatory diseases such as bone bruises, intraosseous ganglions, bone infarcts and even nonspecific cases (2,3). Based on these results BME was assessed on contrast enhanced T1-weighted fat suppressed sequences as it has a higher signal to noise ratio and allowed a shorter scan time for patients. In addition, because T2-weighted fat suppressed sequences could be omitted, coronal sequences of the foot could be added. In total this resulted in a shorter total scan time and more information.

**MRI Scoring**

All MRIs were scored for synovitis, tenosynovitis and BME. Synovitis and BME were scored according to the Outcome Measures in Rheumatology Clinical Trials (OMERACT) rheumatoid arthritis MRI-scoring system (RAMRIS), which was adopted to also include de MTP’s as well (6). Tenosynovitis in the MCP joints, wrist and the MTP joints with available post-contrast sequences was scored as described by Haavaardsholm (7). In MTP joints without post-contrast sequences the median score was imputed. Contrast-enhancement around the extensor tendons in the MCP’s is scored according to this method, but will be referred to as peritendinitis since there is no sheath present (8,9).

MRIs were scored by two readers blinded to clinical data.

In previous research a reference of healthy controls was constituted. Symptom-free volunteers (n = 193) were recruited via advertisements in local newspapers and websites and had no history of inflammatory rheumatic diseases, no joint symptoms during the last month and no evidence of arthritis at physical examination. The age ranged from 19 to 89 years; volunteers were divided into three age groups (18–40 years, n = 51; 40–59 years, n = 90; ≥60 years, n = 52). From these data, percentages were calculated for the prevalence of synovitis, BME and tenosynovitis for different joints, severities and age categories (10).

Synovitis, tenosynovitis and BME scores were compared to the scores of these volunteers in the same location in the matching age-group group (<40, 40-60 and >60) to assess subclinical inflammation. When the scores of two readers differed, the lowest score was compared with the healthy controls. When a score in a location was present in <5% of the volunteers in the same age-group, a location was considered positive for subclinical inflammation.

Synovitis was assessed semi-quantitatively with a range of 0-3 based on the volume of enhancing tissue in the synovial compartment (none, mild, moderate, severe) in 12 joints: MCP 2-5, MTP 1-5 and in three regions of the wrist. Therefore, subclinical inflammation was assessed in 12 joints and counted, resulting in a score of 0-12 locations of subclinical inflammation due to synovitis.

BME was scored on a scale 0-3 based on the affected volume of the bone (no BME, >0-33%, >33-66%, >66%) in the proximal and distal MCP 2-5 (8 bones) and proximal and distal MTP1-5 (10 bones) separately and in 15 bones in the wrist. The CMC-1 joint (Base of meta-carpal 1 and the trapezium) was not included, since it was considered a osteoarthritis location. The proximal and distal MCP2-5 (4 locations) and MTP1-5 (5 locations) were summed into one location, before these locations, and the 13 remaining bones in the wrist, were compared to healthy controls in the same age-group. Consequently, the number of locations of subclinical inflammation due to BME ranged 0-22.

Tenosynovitis was scored semi-quantitatively in 28 tendons, ranging 0-3 based on the thickness of peritendinous effusion or synovial proliferation with contrast enhancement (normal, <2mm, 2-5mm, >5mm): 10 tendons in the wrist and in the flexor and extensor MCP (8 tendons) and MTP tendons (10 tendons), separately. Since 77 patients had no information on tenosynovitis in MTP 1-5, these tendons were not assessed for subclinical inflammation and not included. Thus, subclinical inflammation due to tenosynovitis ranged 0-18.

The number of locations of subclinical inflammation due to synovitis, BME and tenosynovitis were summed, resulting in a number of locations of subclinical inflammation ranging 0-52.

# SUPPLEMENTARY FILE 5

# ICC

Matrix of interreader (A) and intrareader (B) ICC scores of total RAMRIS score of all nine readers who contributed to the data; each MRI was evaluated by two readers.

**A. Interreader ICC**

|  | ***1*** | ***2*** | ***3*** | ***4*** | ***5*** | ***6*** | ***7*** | ***8*** | ***9*** |
| --- | --- | --- | --- | --- | --- | --- | --- | --- | --- |
| ***1*** | *x* | *0.97* | *0.97* | *0.98* | *0.97* | *0.96* | *0.95* | *0.97* | *0.93* |
| ***2*** | *0.97* | *x* | *0.99* | *0.95* | *0.94* | *0.95* | *0.94* | *0.96* | *0.93* |
| ***3*** | *0.97* | *0.99* | *x* | *0.95* | *0.95* | *0.95* | *0.96* | *0.96* | *0.94* |
| ***4*** | *0.98* | *0.95* | *0.95* | *x* | *0.97* | *0.96* | *0.94* | *0.95* | *0.91* |
| ***5*** | *0.97* | *0.94* | *0.95* | *0.97* | *x* | *0.95* | *0.94* | *0.95* | *0.92* |
| ***6*** | *0.97* | *0.95* | *0.95* | *0.96* | *0.95* | *x* | *0.95* | *0.96* | *0.95* |
| ***7*** | *0.95* | *0.94* | *0.96* | *0.94* | *0.94* | *0.95* | *x* | *0.98* | *0.98* |
| ***8*** | *0.97* | *0.96* | *0.96* | *0.95* | *0.95* | *0.96* | *0.98* | *x* | *0.96* |
| ***9*** | *0.93* | *0.93* | *0.94* | *0.91* | *0.92* | *0.95* | *0.98* | *0.96* | *x* |

**B. Intrareader ICC**

| ***1*** | ***2*** | ***3*** | ***4*** | ***5*** | ***6*** | ***7*** | ***8*** | ***9*** |
| --- | --- | --- | --- | --- | --- | --- | --- | --- |
| *0.99* | *0.98* | *0.94* | *0.92* | *0.96* | *0.94* | *0.98* | *0.99* | *0.96* |

# SUPPLEMENTARY FILE 6

# Influence exclusion on coefficients

Patients were included in the Leiden CSA cohort between April 2012 and September 2017. From April 2015 patients with CSA and an MRI positive for the presence of subclinical inflammation could be included in a proof-of-concept trial evaluating the value of DMARD-treatment in CSA (trial registration number: NTR4853). 73 patients that were included in this trial were excluded from follow-up in the observational CSA cohort as part of these patients underwent DMARD-treatment; as the trial is currently ongoing and double blinded, researchers were unware on whom were treated and not treated with DMARDs. Patients included from April 2012-2015 were used as discovery cohort and patients included from April 2015-Sept 2017 as validation cohort. Thus, due to the start of the clinical trial, part of the CSA patients were excluded from the validation cohort and this may not be completely at random, potentially resulting in changed effect sizes.

In this supplementary file we studied the possible influence on the estimated effect sizes.

As shown below, we concluded that when CSA patients with subclinical inflammation were included in the clinical trial and consequently were excluded from follow-up in the observational CSA cohort, this could have resulted in changed effect sizes for risk factors that are studied in the remaining group of CSA patients, when analyzed in the whole group with and without subclinical inflammation (MRI positivity). However this can be solved by performing stratified analyses and comparing risk estimates in patients with and without subclinical inflammation.

This is under the assumptions that inclusion is not associated with the risk factor and the risks estimated within the subgroups with and without subclinical inflammation remain unchanged. These assumptions are presumable if inclusion in the trial is not associated with the studied risk factor or other known arthritis risk factors. Reassuringly, in our cohort it was shown that no major differences exist between patients with a positive MRI that did and did not (want to) participate in the trial and patients that participated (Supplementary file 2).

Presented is an example to illustrate that possible changes in effect sizes can be solved by stratification by MRI positivity:

In this example, we consider a cohort of 400 patients, 200 patients with subclinical inflammation (MRI-positivity) and 200 patients with a negative MRI, and a risk factor was distributed as following. We consider a risk factor that is positively associated with presence of subclinical inflammation, but this does not influence the conclusions:

|  | Subclinical inflammation + | Subclinical inflammation - | **Total** |
| --- | --- | --- | --- |
| Risk factor+ | 80 | 20 | 100 |
| Risk factor- | 120 | 180 | 300 |
| **Total** | 200 | 200 | 400 |

Suppose that the frequency of conversion to arthritis after 1-year for this risk factor is as follows: 40%, 20%, 20% and 10% for the different groups (see blue cells). These rates are chosen arbitrarily, and choosing different rates would not alter our conclusions.

|  | Subclinical inflammation + | Subclinical inflammation - | **Total** |
| --- | --- | --- | --- |
| Risk factor+ | 32/80 (40%) | 4/20 (20%) | 36/100 (36%) |
| Risk factor- | 24/120 (20%) | 18/180 (10%) | 42/300 (14%) |
| **Total** | 56/200 (28%) | 22/200 (11%) | 78/400 (19.5%) |

The blue cells show the conversion rates for all 4 groups of patients: Risk factor+/ Subclinical inflammation +, Risk factor+/ Subclinical inflammation -, Risk factor-/ Subclinical inflammation + and Risk factor-/ Subclinical inflammation-

In the orange cells the total absolute risks of conversion to arthritis in both risk factor groups is shown. It can be seen that the relative risk of the group with risk factor compared to the group without the risk factor is 36%/14% = 2.57.

In the green cells the absolute risks of conversion in both subclinical inflammation groups are shown. Within the group of patients with subclinical inflammation the relative risk of having the risk factor is 40%/20%=2.00 and within the group of patients without subclinical inflammation it is the same: 20%/10%=2.00.

Now part of the patients with subclinical inflammation is excluded from follow-up. Here the assumption is made that this exclusion is independent of the risk factor. In our case, when the risk factors that are considered are findings seen on the MRI, unknown to both patient and doctor, it is presumable that these risk factors do not influence patients in their decision to enter the trial. This would yield the following patient numbers, if we suppose that half of the population of patients with subclinical inflammation is excluded:

|  | Subclinical inflammation + | Subclinical inflammation - | **Total** |
| --- | --- | --- | --- |
| Risk factor+ | 40 | 20 | 60 |
| Risk factor- | 60 | 180 | 240 |
| **Total** | 100 | 200 | 300 |

Importantly, it is assumed that the risk for arthritis development for patients in each of the 4 groups (indicated in blue) remains the same. In our cohort this is presumable since, no major differences were found between included and not-included patients. Then the following risks will be found:

|  | Subclinical inflammation + | Subclinical inflammation - | **Total** |
| --- | --- | --- | --- |
| Risk factor+ | 16/40 (40%) | 4/20 (20%) | 20/60 (33.3%) |
| Risk factor- | 12/60 (20%) | 18/180 (10%) | 16/240 (12.5%) |
| **Total** | 28/100 (28%) | 22/200 (11%) | 50/300 (16.7%) |

As can be seen in the orange cells of the table conversion rates in the total groups with and without the risk factor are both lower (33.3% vs 36% and 12.5% vs 14%). This is due to exclusion of patients with subclinical inflammation but no exclusion of patients without subclinical inflammation. Since subclinical inflammation is associated with increased arthritis development, patients with a higher chance of arthritis were excluded and the arthritis risk in the remaining patients is lower. However, the relative risk for the risk factor is now higher 33.3%/12.5%=2.67 than the situation without exclusion of the patients (2.57).

It can be seen that within the group of patients with subclinical inflammation the conversion rates did not change, because the distribution of the risk factor did not change. As a consequence in this stratum the relative risk remained similar, despite the fact that the total group had reduced in size. Also the conversion rates in the group of patients without subclinical inflammation remained the same, as did the relative risks in this group.

Thus, although in the total patient group the relative risk was changed (in our setting increased), by stratification for the source of selection (in our case subclinical inflammation) unchanged risk estimates were obtained in both subgroups. Hence the latter estimates are comparable to a setting without exclusion due to a trial.

In Cox analysis we corrected for the presence of subclinical inflammation instead of stratifying, by adding it as covariate in the model. An MRI with one of our main predictors (number of affected-locations >0 and MCP-extensor peritendinitis) is always classified as having subclinical inflammation. Therefore, in analyses of these variables, adjustment for subclinical inflammation will have had the same effect as stratification. Moreover, the final results are PPVs, which were calculated stratified for subclinical inflammation: one subgroup for patients without and four subgroups with subclinical inflammation.

Explanation in formulas:

Let A denote Arthritis yes/no, Let M denote MRI-positivity yes/no and let R denote presence of a risk-factor yes/no.


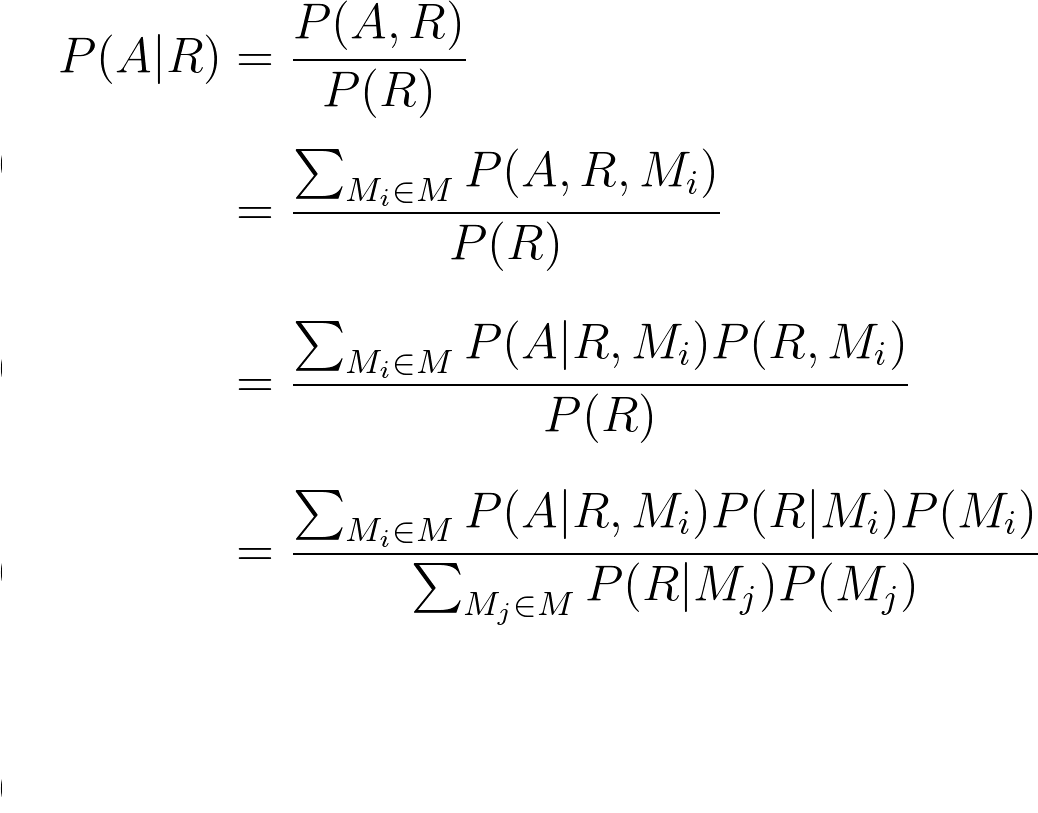


Since P(M) has changed between cohorts due to exclusion of MRI-positive patients, P(A|R) changes, even if P(A|R,M) and P(R|M) do not change.

If we assume that P(A|R,M) is the same in the discovery and the validation set:


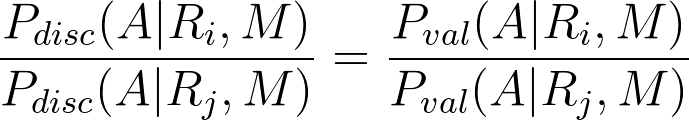


Therefore we expect that the estimated effect sizes do not change after stratification for MRI-positivity.

# SUPPLEMENTARY FILE 7

# Kaplan Meijer curves

Kaplan Meijer curves of conversion to inflammatory arthritis within the discovery cohort. Presented per number of locations (joint/tendon/bone) with subclinical inflammation (A), after categorisation of number of locations in three groups (B), with or without MCP extensor peritendinitis (C), with and without severe subclinical inflammation (D) and with or without inflammation in both the wrist and the MTPs (E).


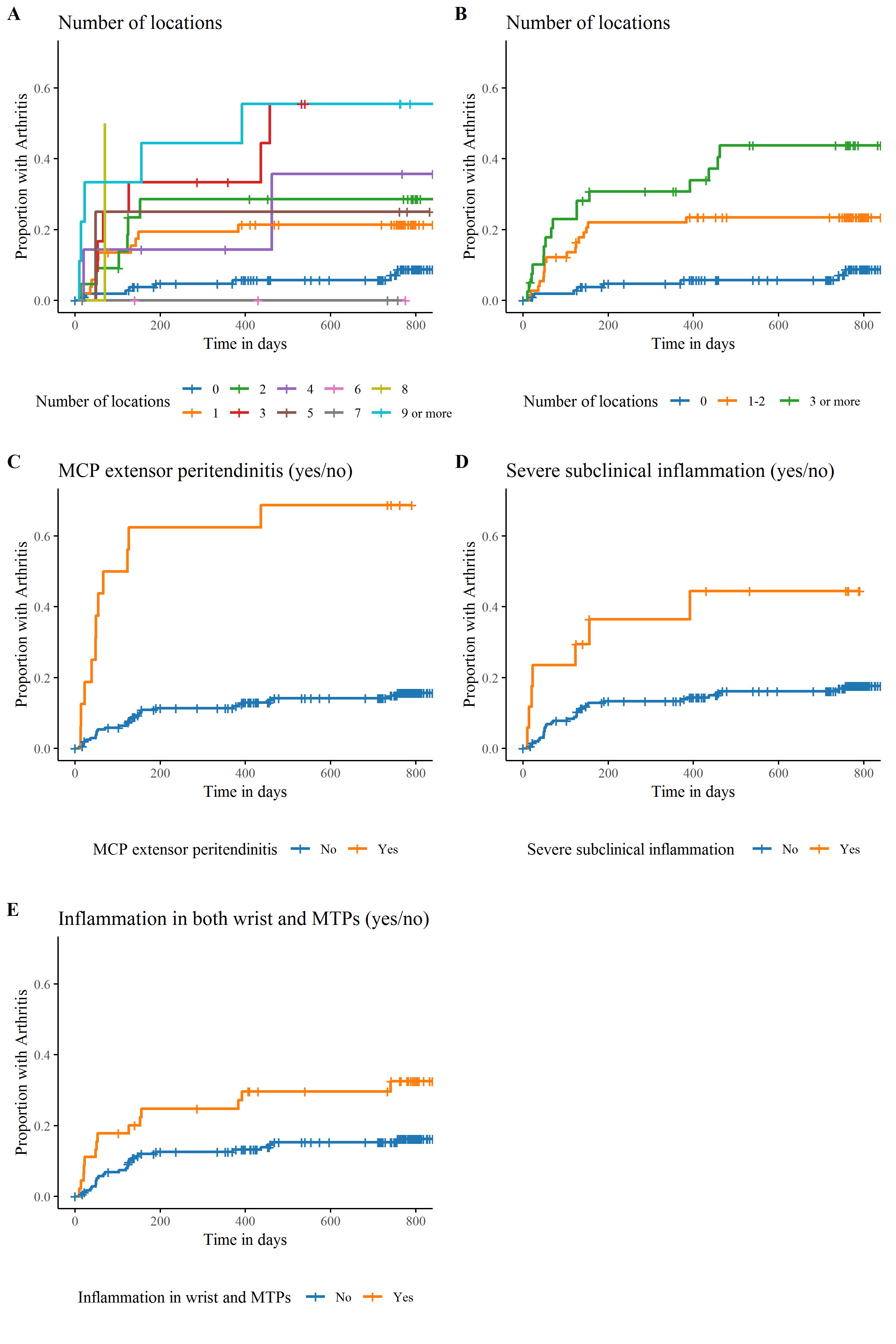


# SUPPLEMENTARY FILE 8

**Results of LASSO regression**

Odds ratios on arthritis development within 1 year of LASSO regression including (A) all inflammatory features (presence yes/no) and (B) all possible combinations of inflammatory features (presence yes/no). Only odds ratios from variables with non-zero coefficients are shown. As is default for LASSO regression, no confidence intervals are shown.

**LASSO regression on all inflammatory features**

| **Feature** | **Odds Ratio** | **Number of patients** |
| --- | --- | --- |
| MCP2 extensor peritendinitis | 3.55 | 6 |
| MCP3 extensor peritendinitis | 2.30 | 9 |
| MCP4 extensor peritendinitis | 1.38 | 8 |

Combinations with this colour are incorporated in the variable “presence of extensor peritendinitis”

**LASSO regression on all possible combinations of inflammatory features**

| **Feature 1** | **Feature 2** | **Odds Ratio** | **Number of patients** |
| --- | --- | --- | --- |
| MCP2 extensor peritendinitis | - | 1.80 | 6 |
| MCP3 extensor peritendinitis | - | 6.11 | 9 |
| MCP3 extensor peritendinitis | MTP5 flexor tenosynovitis | 1.75 | 1 |
| MCP3 extensor peritendinitis | MTP2 flexor tenosynovitis | 1.00 | 1 |
| MCP4 extensor peritendinitis | MCP4 synovitis | 2.05 | 3 |
| MTP1 synovitis | MTP5 proximal BME | 7.46 | 2 |
| MTP1 synovitis | Wrist tenosynovitis V | 5.53 | 3 |
| MTP1 synovitis | Wrist tenosynovitis 2 | 1.02 | 4 |
| MTP1 flexor tenosynovitis | Wrist BME trapezoid | 7.61 | 2 |
| MTP1 flexor tenosynovitis | Wrist intercarpal synovitis | 1.00 | 2 |
| MCP4 synovitis | Wrist BME Scaphoïd | 2.05 | 2 |
| MCP2 flexor tenosynovitis | Wrist BME Ulna | 2.75 | 1 |
| Wrist intercarpal synovitis | Wrist tenosynovitis 4 | 4.39 | 11 |

Combinations with this colour are incorporated in the variable “presence of extensor peritendinitis”

Combinations with this colour are incorporated in the variable “combination of inflammatory lesion in the MTPs and the Wrist”

**Legend:** MCP: metacarpophalangeal; BME: Bone marrow edema; MTP: metatarsophalangeal; Tenosynovitis wrist: (V) extensor digiti quinti proprius (2) ulnar bursa, including flexor digitorum profundus and superficialis tendon quartets; (4) flexor carpi radialis.

# SUPPLEMENTARY FILE 9

# Principal component analysis

**Methods:**

All inflammatory MRI-features (synovitis, BME or tenosynovitis) for every location (joint, bone or tendon) in the discovery cohort were entered in PCA after standardisation to a mean of 0 and a variance of 1. Variables with zero variance were removed. The first two components were plotted and studied with Cox regression.

**Results:**

One variable was removed because of zero variance. The first 2 components explained 15% and 9% of the variance, respectively. The first two components are plotted in below and showed no apparent discrimination. Cox regression showed that PCA-component 1 was predictive for Arthritis development in univariable analyses but was not included in the model after stepwise regression (as shown in main paper Table 2).

**
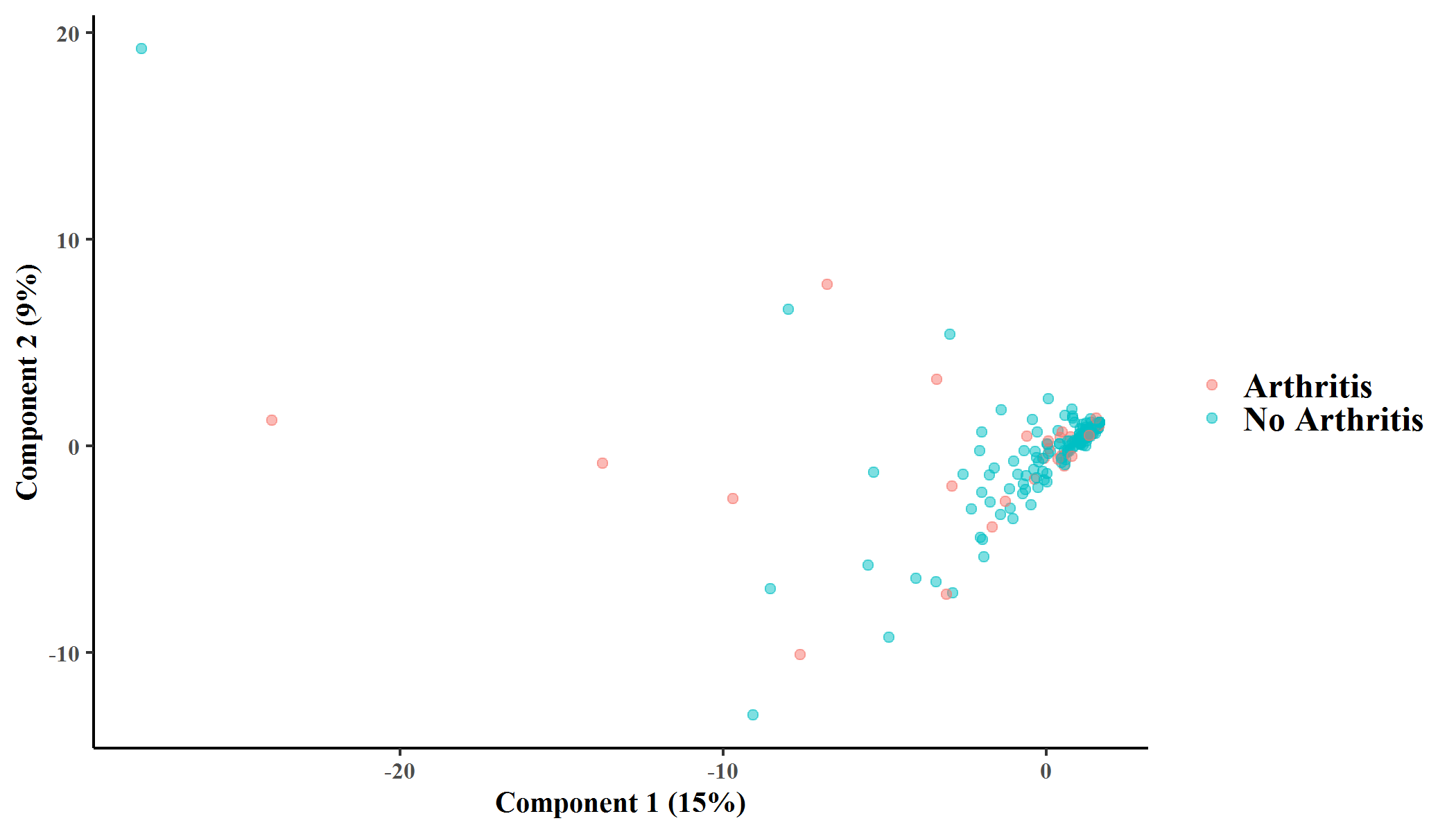
**Figure plotting the first two components of PCA for both patients with and without arthritis after 1-year follow-up in the discovery cohort.

**Legend:** Patients who converted to arthritis within 1-year follow-up are depicted in red and patients that did not convert are depicted in blue. The percentages show how much of the variance of the data is explained by the components.

PCA: Principal component analysis; MRI: Magnetic resonance imaging; BME: Bone marrow edema

# SUPPLEMENTARY FILE 10

**Form to apply risk score**


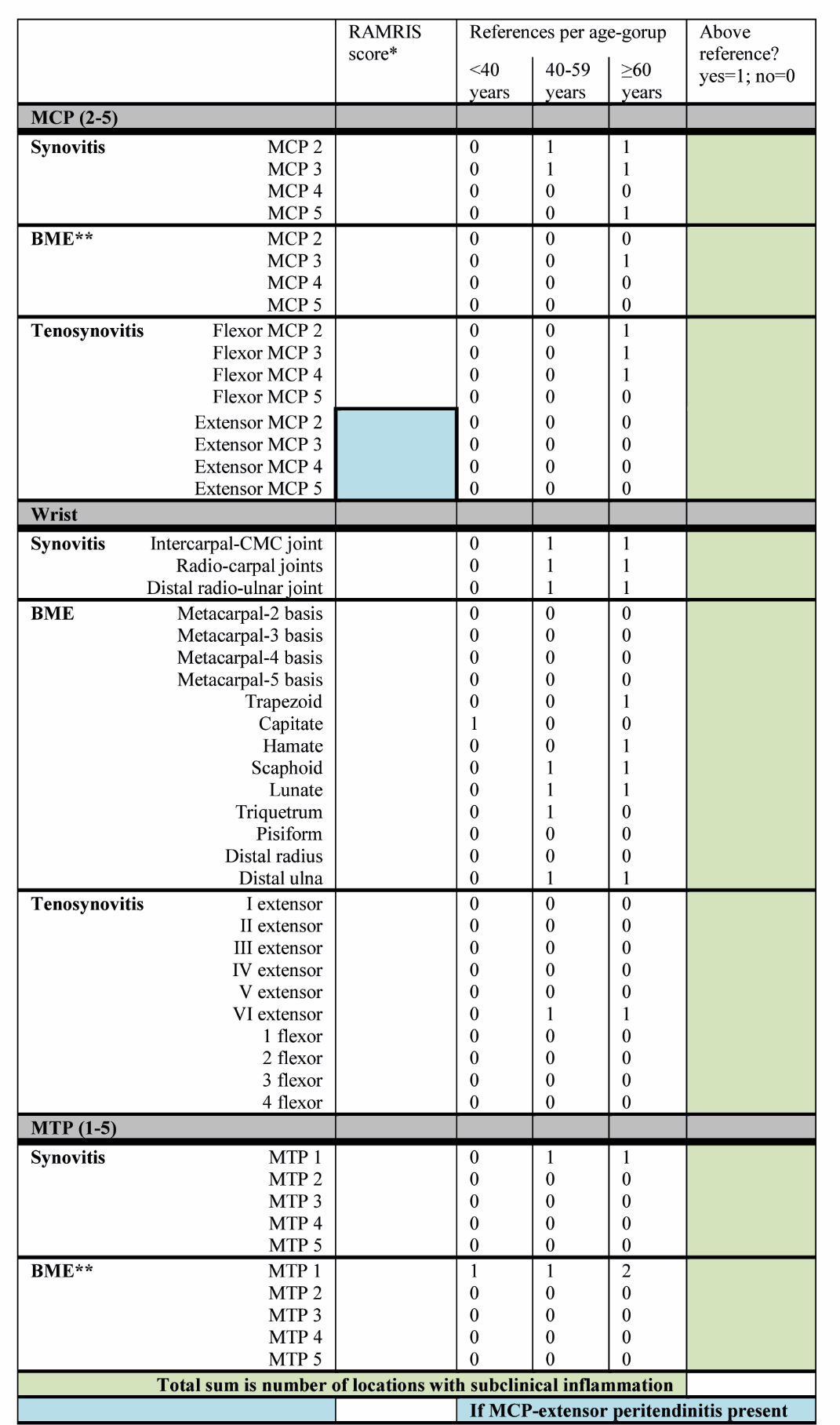
Form to determine the number of locations with subclinical inflammation and presence of MCP-extensor peritendinitis and apply risk score.

**Legend:**

As done previously, subclinical inflammation is scored when both readers score inflammation (synovitis, BME or tenosynovitis) in a location that is present in <5% of the healthy persons in the same age-category at the same location (10-12).

To apply risk score: Sum locations with subclinical inflammation (green column) and identify MCP-extensor peritendinitis (blue). Online version: www.mrincsa.com.

* all MRIs were scored in line with the RAMRIS method, as described in Supplementary file 4.

**Sum BME scores from the proximal and distal and proximal MCPs and MTPs to obtain the BME score for those locations

MCP: metacarpophalangeal; BME: Bone marrow edema; MTP: metatarsophalangeal; Tenosynovitis wrist: (I) extensor pollicis brevis, abductor pollicis longus; (II) extensor carpi radialis brevis, extensor carpi radialis longus; (III) extensor pollicis longus; (IV) extensor digitorum communis, extensor indicus proprius; (V) extensor digiti quinti proprius; (VI) extensor carpi ulnaris; (1) flexor carpi ulnaris; (2) ulnar bursa, including flexor digitorum profundus and superficialis tendon quartets; (3) flexor pollicis longus in radial bursa; (4) flexor carpi radialis.

# SUPPLEMENTARY FILE 11


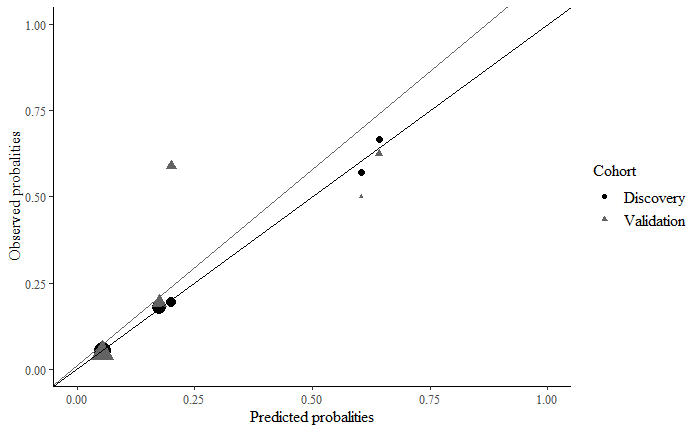
**Calibration plot of predicted and observed probabilities in the discovery and validation coho**rt.

**Legend:** In this calibration plot the PPVs predicted by logistic regression in the discovery cohort in the five risk groups are compared with the observed probabilities in the two cohorts. The calibration intercept in the discovery cohort was 0.00 (95% Confidence interval 0.06-0.06) and the slope 1.00 (0.70-1.30). The calibration intercept in the validation cohort was 0.01 (95% Confidence interval 0.05-0.07) and the slope 1.14 (0.80-1.48). The size of the figures depict the number of patients in each group.

# SUPPLEMENTARY FILE 12

**AUC curves of the in the derivation and validation cohorts and of a model that only considered MRI-positivity.**

**Legend:** The AUC of the model incorporating number of locations and presence of MCP-extensor peritendinitis was 0.74 (95% Confidence Interval 0.65-0.84) in the discovery cohort and 0.81 (95% Confidence Interval 0.72-0.90) in the validation cohort. Model that only considered MRI-positivity and ‘MRI-negativity’ had an AUC of 0.69 (95% Confidence interval 0.60-0.78) in discovery cohort.

# SUPPLEMENTARY FILE 13

**Sensitivity analysis with the outcome inflammatory arthritis within 2 years**


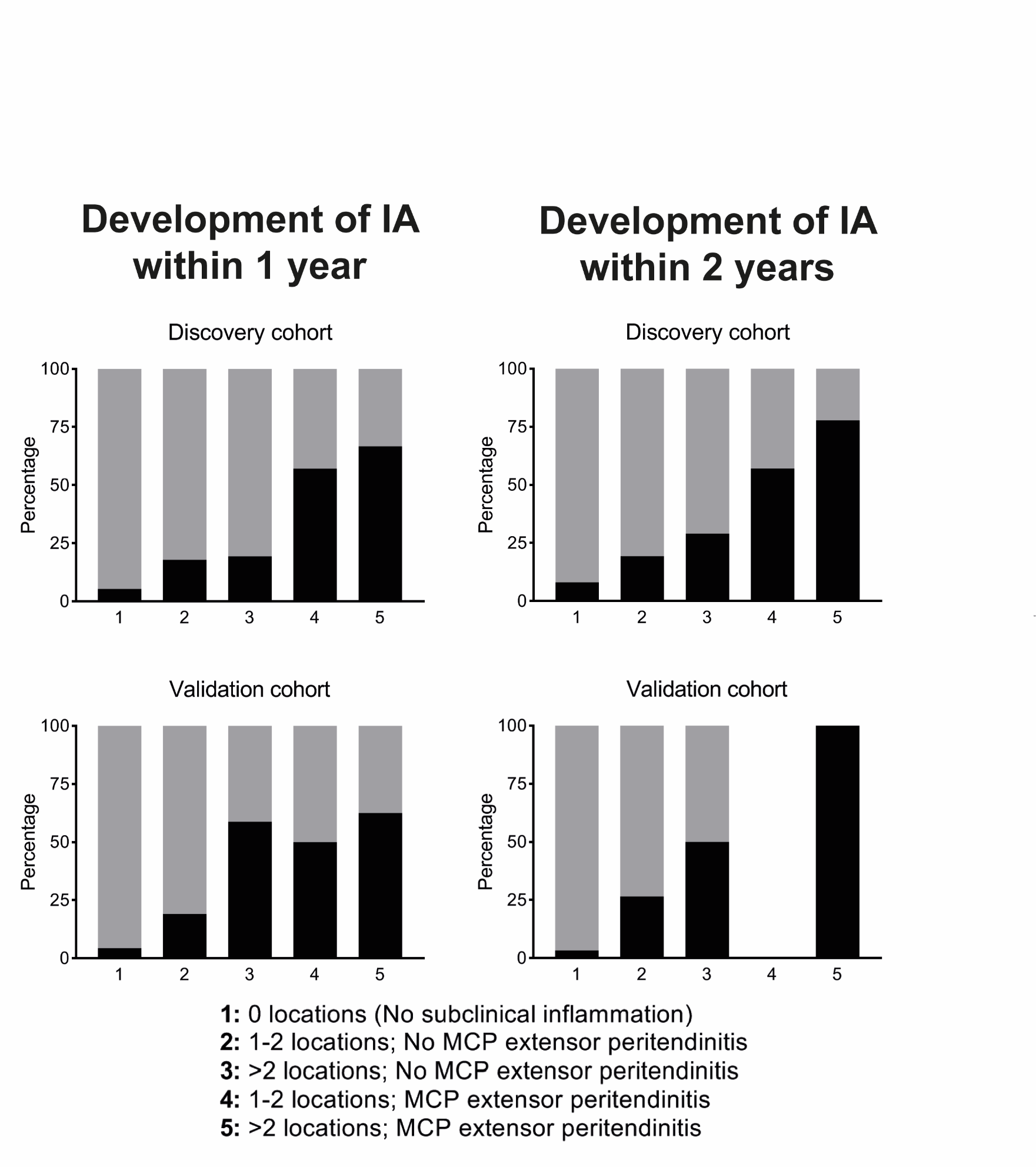
Proportion of patients that that developed arthritis (PPVs in black) in the first year (also shown in Figure 3 in the main paper) and in the first two years per risk category in the discovery and validation cohorts.

**Legend:**

IA: clinically apparent Inflammatory Arthritis; locations: number of locations with subclinical inflammation.

**Upper left graph:** Positive predictive values on IA in the discovery cohort within 1 year; No subclinical inflammation (5% (95% Confidence interval 3%-11%, n=111), 1-2 locations (18% (11%-29%), n=67) or ≥3 locations (19% (9%-36%), n=31) with subclinical inflammation but without MCP-extensor peritendinitis; and 1-2 locations (57% (25%-84%), n=7) or ≥3 locations (67% (35%-88%), n=9) with MCP-extensor peritendinitis.

**Upper right graph:** Positive predictive values on IA in the discovery cohort within 1 year; No subclinical inflammation (8% (95% Confidence interval 4%-15%, n=111), 1-2 locations (19% (12%-30%), n=67) or ≥3 locations (29% (16%-47%), n=31) with subclinical inflammation but without MCP-extensor peritendinitis; and 1-2 locations (57% (25%-84%), n=7) or ≥3 locations (78% (45%-94%), n=9) with MCP-extensor peritendinitis.

**Lower left graph:** Positive predictive values on IA in the validation cohort within 1 year; No subclinical inflammation (4% (95% Confidence interval 2%-9%, n=135), 1-2 locations (19% (10%-33%), n=47) or ≥3 locations (59% (35%-78%), n=17) with subclinical inflammation but without MCP-extensor peritendinitis; and 1-2 locations (50% (3%-97%), n=2) or ≥3 locations (63% (31%-86%), n=8) with MCP-extensor peritendinitis.

**Lower right graph:** Positive predictive values on IA in the validation cohort within 2 years; No subclinical inflammation (3% (95% Confidence interval 1%-9%, n=91), 1-2 locations (26% (15%-43%), n=34) or ≥3 locations (50% (27%-73%), n=14) with subclinical inflammation but without MCP-extensor peritendinitis; and 1-2 locations (n=0) with MCP-extensor peritendinitis or ≥3 locations (100% (44%-100%), n=3) with MCP-extensor peritendinitis.

# SUPPLEMENTARY FILE 14

**Sub-analyses within CSA patients that also fulfilled the EULAR definition**


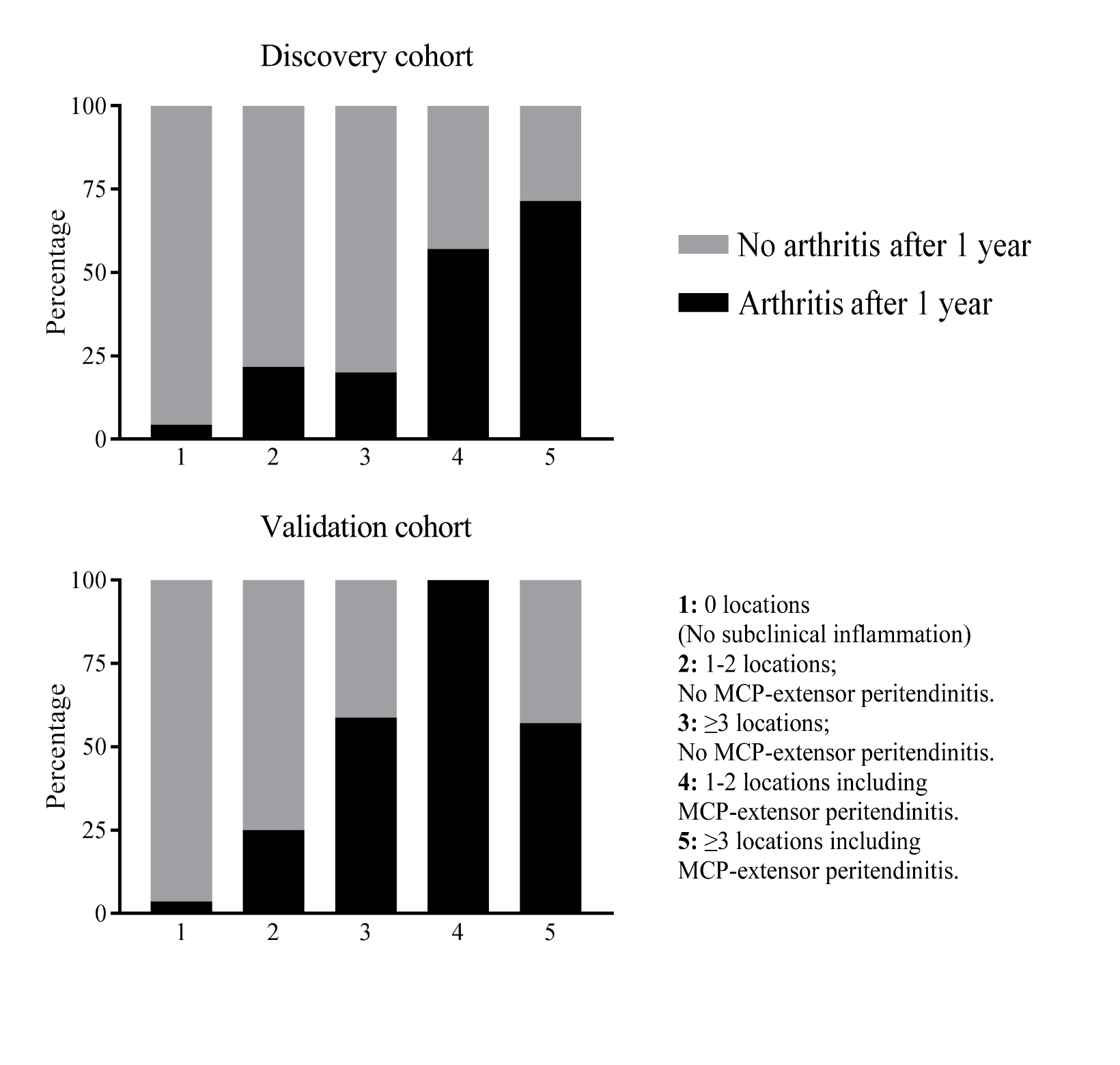
Proportion of patients that fulfilled the EULAR definition for CSA that developed arthritis in the first year (PPVs) per risk category in the discovery and validation cohorts.

**Upper graph:** Positive predictive values in the discovery cohort; fulfilling the EULAR definition for CSA: No subclinical inflammation (4% (95% C.I. 2%-12%, n=68), 1-2 locations (22% (12%-36%), n=46) or ≥3 locations (20% (9%-39%), n=25) with subclinical inflammation but without MCP-extensor peritendinitis; and 1-2 locations (57% (25%-84%), n=7) or ≥3 locations (71% (36%-92%),n=7) with MCP-extensor peritendinitis.

**Lower graph:** Positive predictive values in the validation cohort, cohort; fulfilling the EULAR definition for CSA: No subclinical inflammation (4% (95% C.I. 1%-10%, n=83), 1-2 locations (25% (13%-43%), n=28) or ≥3 locations (58% (32%-81%), n=12) with subclinical inflammation but without MCP-extensor peritendinitis; and 1-2 locations (100% (5%-100%), n=1) or ≥3 locations (57% (25%-84%),n=7) with MCP-extensor peritendinitis.

**REFERENCES**

1. Stomp W, Krabben A, van der Heijde D, Huizinga TW, Bloem JL, van der Helm-van Mil AH, et al. Aiming for a shorter rheumatoid arthritis MRI protocol: can contrast-enhanced MRI replace T2 for the detection of bone marrow oedema? Eur Radiol. 2014;24(10):2614-22.

2. Schmid MR, Hodler J, Vienne P, Binkert CA, Zanetti M. Bone marrow abnormalities of foot and ankle: STIR versus T1-weighted contrast-enhanced fat-suppressed spin-echo MR imaging. Radiology. 2002;224(2):463-9.

3. Mayerhoefer ME, Breitenseher MJ, Kramer J, Aigner N, Norden C, Hofmann S. STIR vs. T1-weighted fat-suppressed gadolinium-enhanced MRI of bone marrow edema of the knee: computer-assisted quantitative comparison and influence of injected contrast media volume and acquisition parameters. J Magn Reson Imaging. 2005;22(6):788-93.

4. Tamai M, Kawakami A, Uetani M, Fukushima A, Arima K, Fujikawa K, et al. Magnetic resonance imaging (MRI) detection of synovitis and bone lesions of the wrists and finger joints in early-stage rheumatoid arthritis: comparison of the accuracy of plain MRI-based findings and gadolinium-diethylenetriamine pentaacetic acid-enhanced MRI-based findings. Mod Rheumatol. 2012;22(5):654-8.

5. Sudol-Szopinska I, Jurik AG, Eshed I, Lennart J, Grainger A, Ostergaard M, et al. Recommendations of the ESSR Arthritis Subcommittee for the Use of Magnetic Resonance Imaging in Musculoskeletal Rheumatic Diseases. Semin Musculoskelet Radiol. 2015;19(4):396-411.

6. Ostergaard M, Peterfy C, Conaghan P, McQueen F, Bird P, Ejbjerg B, et al. OMERACT Rheumatoid Arthritis Magnetic Resonance Imaging Studies. Core set of MRI acquisitions, joint pathology definitions, and the OMERACT RA-MRI scoring system. J Rheumatol. 2003;30(6):1385-6.

7. Haavardsholm EA, Ostergaard M, Ejbjerg BJ, Kvan NP, Kvien TK. Introduction of a novel magnetic resonance imaging tenosynovitis score for rheumatoid arthritis: reliability in a multireader longitudinal study. Ann Rheum Dis. 2007;66(9):1216-20.

8. Kurppa K, Waris P, Rokkanen P. Peritendinitis and tenosynovitis. A review. Scand J Work Environ Health. 1979;5 suppl 3:19-24.

9. Doyle JR, Botte MJ. Surgical anatomy of the hand and upper extremity. Philadelphia: Lippincott, Williams & Wilkins; 2003. 783 p.

10. Mangnus L, van Steenbergen HW, Reijnierse M, van der Helm-van Mil AH. Magnetic Resonance Imaging-Detected Features of Inflammation and Erosions in Symptom-Free Persons From the General Population. Arthritis Rheumatol. 2016;68(11):2593-602.

11. Boer AC, Burgers LE, Mangnus L, Ten Brinck RM, Nieuwenhuis WP, van Steenbergen HW, et al. Using a reference when defining an abnormal MRI reduces false-positive MRI results-a longitudinal study in two cohorts at risk for rheumatoid arthritis. Rheumatology (Oxford). 2017;56(10):1700-6.

12. van Steenbergen HW, van Nies JA, Huizinga TW, Reijnierse M, van der Helm-van Mil AH. Subclinical inflammation on MRI of hand and foot of anticitrullinated peptide antibody-negative arthralgia patients at risk for rheumatoid arthritis. Arthritis Res Ther. 2014;16(2):R92.
